# Exploring the Preventive Effect and Mechanism of Senile Sarcopenia Based on “Gut–Muscle Axis”

**DOI:** 10.3389/fbioe.2020.590869

**Published:** 2020-11-05

**Authors:** Xiaoshan Liao, Mengting Wu, Yuting Hao, Hong Deng

**Affiliations:** Guangdong Provincial Key Laboratory of Tropical Disease Research, Department of Nutrition and Food Hygiene, School of Public Health, Southern Medical University, Guangzhou, China

**Keywords:** age-related sarcopenia, gut microbiota, gut-muscle axis, mechanisms, therapy

## Abstract

Age-related sarcopenia probably leads to chronic systemic inflammation and plays a vital role in the development of the complications of the disease. Gut microbiota, an environmental factor, is the medium of nutritional support to muscle cells, having significant impact on sarcopenia. Consequently, a significant amount of studies explored and showed the presence of gut microbiome–muscle axis (gut–muscle axis for short), which was possibly considered as the disease interventional target of age-related sarcopenia. However, a variety of nutrients probably affect the changes of the gut–muscle axis so as to affect the healthy balance of skeletal muscle. Therefore, it is necessary to study the mechanism of intestinal–muscle axis, and nutrients play a role in the treatment of senile sarcopenia through this mechanism. This review summarizes the available literature on mechanisms and specific pathways of gut–muscle axis and discusses the potential role and therapeutic feasibility of gut microbiota in age-related sarcopenia to understand the development of age-related sarcopenia and figure out the novel perspective of the potential therapeutic interventional targets.

## Introduction

With the global improvement of life quality and medical care, average human life expectancy has be prolonged dramatically and it will prolong continuously (Kontis et al., 2017; Ben-Haim et al., 2018; Partridge et al., 2018). However, age-related diseases are thriving (Carmona and Michan, 2016), which means longer human lives result in a global burden of late-life disease (Partridge et al., 2018). It is imperative to obtain more understanding about the aging process, since healthy aging has become a popular topic (Lu et al., 2019). In recent years, more and more studies suggest that the muscle mass and function significantly and inexorably decline with age (Blau et al., 2015; Curtis et al., 2015; Brook et al., 2016; Cartee et al., 2016; Giallauria et al., 2016; Francis et al., 2017; Tezze et al., 2017; Tieland et al., 2018; Wilkinson et al., 2018; Larsson et al., 2019), which is termed as *sarcopenia* (Larsson et al., 2019). Sarcopenia is strictly defined as an age-related syndrome, which is characterized by progressive and generalized loss of skeletal muscle mass and strength. Moreover, sarcopenic obesity, a new concept that emerged recently, reflects a combination of sarcopenia and obesity (Choi, 2016). Sarcopenia may reduce mobility lead to fall-related injuries, such as bone fracture, diminish health-related quality of life and lead to premature death (Curtis et al., 2015; Shaw et al., 2017; Larsson et al., 2019). A systematic review and meta-analysis study shows that a substantial proportion of the old people have sarcopenia, and the overall estimate of prevalence in both men and women is 10% (Shafiee et al., 2017). The cause of sarcopenia or sarcopenia obesity is complex. The lack of exercise, age-related decreases in hormone concentrations and low vitamin D status are considered as the risk factors inducing sarcopenia presently (Dhillon and Hasni, 2017; Petroni et al., 2019). Interestingly, an increasing number of studies suggest gut microbiota is closely associated with sarcopenia in aging (Ticinesi et al., 2019b).

It is not difficult to find that the relation between the gut microbiota and human health is being increasingly recognized, so the concept that the human gut microbiota is involved in multiple interactions influencing host health during the host’s entire lifespan is well known. Notably, the composition of human gut microbiota changes with age, and some transition points and patterns in the changes of composition in gut microbiota with age have been indicated (Odamaki et al., 2016). A separate phylogenetic study finds out the core intestinal flora of people in different age groups: young people (22–48 years old), elderly (65–75 years old), centenarians (99–104 years old), and half-centenarians (105–109 years old; Biagi et al., 2016). The core intestinal flora is composed of *Bacteroides*, Rumen bacteria, and Spirulina, implying unhealthy intestine status probably due to changes of composition of the core intestinal flora or the risk of unhealthy intestines with age. Actually, as the biological age increases, the overall abundance of gut microbiome decreases, with the addition of some microbial classifications related to unhealthy aging (Kim and Jazwinski, 2018). Compared with younger individuals, the frail elderly have a greater change in the intestinal flora, with an observed change that dominated population of *Bacteroides* in the microbial community (Mangiola et al., 2018). The transition between the adults and the elderly is mainly characterized by a decrease in bacterial diversity, the transition between the adults and the elderly is mainly characterized by a decrease in bacterial diversity and a decrease in bifidobacteria and an increase in *Clostridium*, *Lactobacillus*, *Enterobacteriaceae*, and *Enterococcus* (Mitsuoka, 2014). A study shows that the fecal samples of individuals from 0 to 104 years old were analyzed by 16S rRNA sequencing, whose results support that the gut microbiome changes with age (Odamaki et al., 2016).

Specifically speaking, the composition of gut microbiota is proved to be dynamic throughout the lifespan attributing to the factors such as dietary changes, antibiotic intake, age, disease, and so on (Manuel Marti et al., 2017). The host and the microbiota are extremely related, owing to the functions mediated or affected by bacteria of host. The regulatory effect of the microbiota for the balance of body health includes fiber catabolism, host immune system regulation and resistance to pathogens, vitamin and amino acid biosynthesis, xenobiotic detoxification, etc. (Kundu et al., 2017). Because of the regulatory effect of gut microbiome on human metabolism and immunology, the gut microbiome is considered as a possible determinant of healthy aging (Claesson et al., 2012; Candela et al., 2014). The maintenance of host microbial homeostasis probably counteracts inflammation (Biagi et al., 2010), intestinal barrier permeability (Nicoletti, 2015), and so on. However, the composition of gut microbiota changes rapidly again, leading to gastrointestinal dysbiosis, which is associated with increasing biological age (Maffei et al., 2017; Salazar et al., 2017). An increasing number of evidence suggest that dysbiosis in the microbiome is associated with a variety of diseases, including atherosclerosis, hypertension, obesity, diabetes (types 1 and 2), cancer, sarcopenia, and so on (Lloyd-Price et al., 2016; Cigarran Guldris et al., 2017; Lau et al., 2017; Li et al., 2017; Rajagopala et al., 2017; Tang et al., 2017; Weiss and Hennet, 2017; Picca et al., 2018a; Durazzo et al., 2019).

Interestingly, although the dysbiosis in the microbiome can induce a lot of diseases, the health effects of regulating gut microbiota in the body obtained more and more attention in recent years. As for sarcopenia, it is remarkable that an increasing number of studies suggest the presence of gut–muscle axis, indicating the gut microbiome may affect the health of skeletal muscle and vice versa (Picca et al., 2018a). A Chinese cohort survey finds that different dietary habits attributed to different geographical locations lead to significant differences in the composition of the gut microbiome (Zhang et al., 2015). Because aging is often accompanied by a decrease in the amount and diversity of fiber-containing foods intake, and a risk of malnutrition and lifestyle, especially diet, plays a vital role on aging gut health (Claesson et al., 2012). Therefore, changes in dietary patterns, supplementation of nutrients, and intervention of active substances improve the incidence of sarcopenia via the gut–muscle axis and have become some of the new treatment methods in recent years.

This review summarized the available literature on evidence and mechanisms of gut–muscle axis and aimed at coming up with promising preventive and therapeutic measures targeting gut microbiome on sarcopenia in order to improve the quality of life in aging and decrease global burden of late-life disease.

## Skeletal Muscle and Microbiome

In recent years, more and more studies have shown that gut microbes are related to skeletal muscle metabolism. The state of gut microbes possibly affects the content and function of skeletal muscle. Intestinal microbial disorders cause health loss to patients and even the elderly, affecting the quality of life. Therefore, it is urgent for us to efficiently find out intervention approach via clarifying the role of the gut microbiota in skeletal muscle metabolism.

### Effect of Gut Microbiome on Skeletal Muscle: Animal Studies

Skeletal muscle descending size and function are related to metabolic disorders (Kelley et al., 1999) and osteoporosis (Nair, 2005), and animal studies show that intestinal microbial metabolism can regulate skeletal muscle function. The study by Honglin Yan et al. finds that, compared with lean Yorkshire pigs, obese Rongchang pigs (RP) have a different composition of gut microbes. The intestinal microbes of RP were transplanted to germ-free (GF) mice, and the muscle characteristics of GF mice are highly similar to those of RP, which indicated gut microbiome plays a potential role in the skeletal muscle development (Yan et al., 2016). Similarly, it is generally believed that the physiological metabolism of gut microbiome, muscle, and immunity is related to age. Combined with the analysis of intestinal microbiome, biochemical indicators of muscle metabolism, and serum proteomics and liposome profile of elderly rats with age, the results show that old rats have a higher inflammation/immune status compared with adult rats; the intestinal flora probably participated in the metabolic processes of musculoskeletal system, nutrition, and inflammation/immunity through various complex mechanisms (Siddharth et al., 2017). The antibiotic metronidazole-treated mice have a significant increase of the bacterial phylum *Proteobacteria* in fecal pellets, accompanied by a decrease in muscle weight of the hind limbs, resulting in smaller tibial anterior muscle fibers. In the gastrocnemius muscle, metronidazole treatment leads to the increasing expression of neurogenic atrophy-related biochemical indicators of skeletal muscle, including the up-regulation of HDAC4, myogenin, MuRF1, and avergin1 (Manickam et al., 2018).

Based on the physiological analytic results, intestinal microbial composition is closely related to skeletal muscle content and related biochemical and metabolic indicators (Manickam et al., 2018; Lahiri et al., 2019); some researchers have tried to apply intestinal flora transplantation in skeletal muscle improvement. Certain gut microbiomes are able to produce intestinal metabolites that promote skeletal muscle anabolism. Feces transplanted from unhealthy children (or malnourished children) changed the growth of mice (Ticinesi et al., 2017). Having a gut microbial composition similar to that of the donor, the sterile mice transplanted with feces from the elderly show high (with higher lean meat and lower fat mass) or low function (Fielding et al., 2019). The high function transplanted mice show higher grip strength and a higher proportion of microorganisms. GF mice had lower muscle mass and fewer muscle fibers, while muscle atrophy markers increased compared to pathogen-free mice; however, these are mostly reversed after fecal transplantation and short-chain fatty acid (SCFA) treatment, surprisingly (Lahiri et al., 2019). Researchers continuously find from animal models that change of the composition of gut microbiome regulates the metabolic function of skeletal muscle. Therefore, it is of significance to understand the complex relationship between gut microbiome and host physiology to determine approaches of lifestyle/nutrition/pharmaceutical intervention in maintaining the potential range of skeletal muscle health.

### Effect of Gut Microbiome on Skeletal Muscle: Human Studies

The relationship between the composition of the gut microbiome of the elderly and the function of skeletal muscle has also been found in human studies. Changes in gut microbiome that occur when the elderly were transferred to long-term care facilities may eventually change bones and body composition, leading to sarcopenia, osteoporosis, and obesity, increasing the risk of fractures (Inglis and Ilich, 2015). According to the potential close relationship between intestinal population composition and skeletal muscle metabolism, some researchers have explored its possibility as a clinical diagnostic indicator in the clinic. The Sequential and Orthogonalized Covariance Selection (SO-CovSel) prediction model was established by a multimarker method of labeling biomarkers of gut microbial profile, systemic inflammation, and metabolic characteristics in the available cohort of elderly people, and the model correctly distinguished 91.7% of the elderly in the physical frailty and sarcopenia (PF&S) group and 87.5% of the non-PF&S control group (Picca et al., 2020), suggesting close relationship between the gut microbiome, inflammation, nutritional, metabolic status and muscle rebuilding.

Simultaneously, gut microbiome has been explored its possibility as a therapeutic intervention target of skeletal muscle metabolism in the clinic. Sarcopenia, as a common complication in patients with chronic kidney disease (CKD), is related to the activation of protein breakdown signal pathways. The dynamic balance symbiosis system composed of gut microbiome and the human body is destroyed in CKD state, and the resulting intestinal microecological imbalance can accelerate the progress of sarcopenia (Tang et al., 2020). Therefore, the dynamic balance of gut microbiome is considered as the novel and effective intervention target. Clinically, non-target comprehensive metabolomics analysis was performed on middle-aged men with metabolic syndrome treated with resveratrol (RSV). Among men treated with RSV, muscle renewal biochemical markers increase; lipid metabolism is affected; and the urinary derivatives of aromatic amino acids, that mainly reflect the changes of the composition of gut microbiome, have been altered, which may be owing to the change of metabolic function of gut microbiome (Korsholm et al., 2017). The curative effect of the treatment of sarcopenia that targets gut microbiome for intervention has begun to be explored. We will describe the mechanism of action of “gut–muscle axis” and intervention treatment in detail in the following sections.

## Mechanism of Gut-Muscle Axis

**FIGURE 1:**
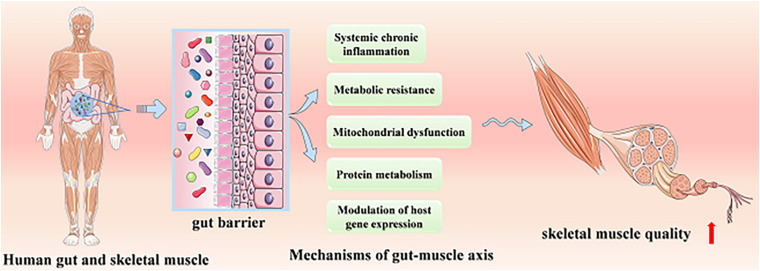
The possible mechanisms of the skeletal muscular improving quality via gut–muscle axis.

At present, the role of the gut–muscle axis in regulating age-related muscle health has been confirmed by animal models and human studies, while its mechanisms have not been systematically understood. We will systematically introduce the mechanisms probably involved, including protein metabolism, systemic chronic inflammation and metabolic resistance, mitochondrial dysfunction, and modulation of host gene expression (Figure 1).

### Protein Metabolism

In fact, gut microbiota can be regarded as a highly significant metabolic organ or endocrine organ that generates bioactive metabolites and impacts physiological processes those are vital to host health such as regulation of immune mediators, energy homeostasis, neurobehavioral development, and gut epithelial health (Marchesi et al., 2016; Takiishi et al., 2017; Tang et al., 2017; Barko et al., 2018). Besides, gut microbiota can alter the bioavailability of amino acids through utilizing several amino acids that originate from both alimentary and endogenous proteins, influencing muscle protein synthesis and breakdown, and having an effect on host muscle (Neis et al., 2015; Karlund et al., 2019). As we all know, protein is vital for skeleton muscle. In addition, the gut microbiota is capable of synthesizing some nutritionally essential amino acids *de novo*, such as tryptophan, which represents the fundamental substrates for muscle protein anabolism (Lin et al., 2017). Tryptophan probably plays a role of stimulating the insulin-like growth factor 1/p70s6k/mTOR pathway in muscle cells and promoting the expression of genes involved in myofibrillar synthesis (Dukes et al., 2015). However, it is also suggested that protein-enriched diets may switch bacterial metabolism toward amino acids degradation and fermentation (Picca et al., 2020).

### Systemic Chronic Inflammation and Metabolic Resistance

It is recognized that age-related sarcopenia can be caused by systemic chronic inflammation, as well as metabolic resistance in aging, and the mechanism is gradually understood by people.

### Systemic Chronic Inflammation and Gut Barrier Function

Studies suggest that age-related systemic chronic inflammation (“inflaming”) is involved in the development of sarcopenia (Boirie, 2009; Bindels and Delzenne, 2013; Ogawa et al., 2016; Steves et al., 2016; Ticinesi et al., 2017; Grosicki et al., 2018; Liguori et al., 2018; Ni Lochlainn et al., 2018). The changes in the gut microbiome could alter the inflammatory state of the individual and consequently result in sarcopenia (Biagi et al., 2010; Bindels and Delzenne, 2013; Quigley, 2013; Steves et al., 2016; Ni Lochlainn et al., 2018; Picca et al., 2018a). It is reported that modulation of the gut microbiota can influence the gut’s barrier function, thereby playing an important role in maintaining the balance of proinflammatory and anti-inflammatory responses (Ni Lochlainn et al., 2018; Picca et al., 2018a). The healthy gut microbiome induces a big variety of host responses within the intestinal mucosa and thereby strengthens the gut barrier function, exerting immunomodulatory actions within the gut and beyond (Bindels and Delzenne, 2013; Lin and Zhang, 2017; Ni Lochlainn et al., 2018). In addition, work in animal models shows evidence of gut barrier dysfunction in association with age-associated microbial dysbiosis, increasing intestinal permeability (Grosicki et al., 2018; Ni Lochlainn et al., 2018; Thevaranjan et al., 2018). This could contribute to facilitating translocation of microbial byproducts into the circulation. Microbial byproducts include endotoxins such as lipopolysaccharide (LPS); they can induce systemic chronic inflammation as well as insulin resistance which is one form of metabolic resistance and finally lead to sarcopenia (Bindels and Delzenne, 2013; Ni Lochlainn et al., 2018). A low representation of SCFAs, producers in gut microbiota, are proved involved in increasing subclinical chronic inflammation and then resulted in sarcopenia (den Besten et al., 2013; Ticinesi et al., 2017). In other words, SCFAs produced by gut microbiota can help reduce inflammation and thereby prevent sarcopenia. From a skeletal muscle perspective, one of the most studied mediators among SCFAs is butyrate. Butyrate also plays a vital role in intestinal barrier function and therefore may be associated in intestinal permeability (Peng et al., 2009; Ni Lochlainn et al., 2018). Besides, its anti-inflammatory properties are demonstrated in inflammatory bowel diseases (Leonel and Alvarez-Leite, 2012; Ticinesi et al., 2017). Interestingly, lack of SCFAs can also lead to metabolic resistance (Sonnenburg and Backhed, 2016; Ticinesi et al., 2017; Vaiserman et al., 2017; Ni Lochlainn et al., 2018).

### Metabolic Resistance

**FIGURE 2:**
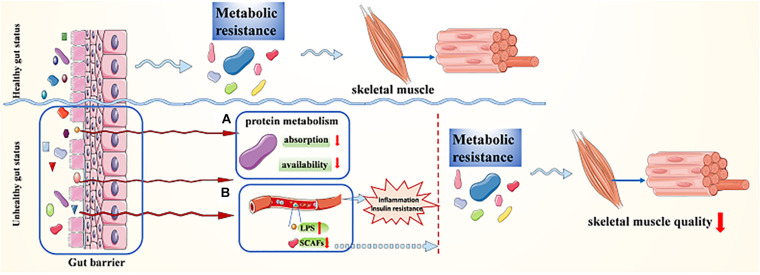
Transmutation of gut status maintains sarcopenia via metabolic resistance. **(A)** The changes in gut microbiome composition and/or diversity result in changes in protein metabolism; **(B)** gut microbial dysbiosis leads to gut barrier dysfunction, which causes the concentration of LPS in the blood to rise and/or SCFAs to decline.

It is suggested that age-related sarcopenia is closely associated with metabolic resistance (Figure 2; Ticinesi et al., 2017; Ni Lochlainn et al., 2018). There is evidence showing “anabolic resistance” in older adults, which means a higher dose of protein is needed to achieve the same myofibrillar protein synthesis response as a younger person (Dillon, 2013; Welch, 2014; Moore et al., 2015; Mitchell et al., 2017). The gut microbiota is involved in many of the postulated mechanisms for anabolic resistance in older people, either directly or indirectly, and it is probably to be a complex interaction between these postulated processes (Ni Lochlainn et al., 2018). The changes in gut microbiome composition and/or diversity result in changes in protein metabolism (Figure 2A), including absorption as well as availability reduction and increased hydrolysis, leading to anabolic resistance, reduction of muscle protein synthesis, and the development of sarcopenia (Rampelli et al., 2013; Mitchell et al., 2017). Besides, gut microbial dysbiosis can lead to gut barrier dysfunction (Figure 2B), which causes the concentration of LPS in the blood to rise. In addition, gut microbial dysbiosis can also result in reduced production of SCFAs. Both the rise of LPS concentration in the blood and the reduction of SCFAs can bring about inflammation and insulin resistance first and then lead to metabolic resistance, or they can also result in metabolic resistance without causing inflammation or insulin resistance first (Ni Lochlainn et al., 2018; Mesinovic et al., 2019). Anabolic resistance should not be limited to one or two mechanisms but must be regarded as a complex and multidimensional construct. The etiologies and mechanisms implicated are not clear; thus, further investigation is required to understand the potential role of the gut microbiota in a variety of postulated mechanisms for anabolic resistance.

### Mitochondrial Dysfunction

In skeletal muscle, primary aging leads to mitochondrial energetics deficiency and muscle mass reduction (Cartee et al., 2016). It is suggested that ultrastructural modifications in the number and functionality of mitochondria caused reduced muscle protein synthesis (Marzetti et al., 2013, 2016; St-Jean-Pelletier et al., 2017). Ibebunjo et al. suggest that aged rats lose muscle mass and function gradually (i.e., sarcopenia) through mechanisms involving mitochondrial dysfunction (Ibebunjo et al., 2013; Siddharth et al., 2017). As for human, a study finds that sedentary but not active humans show an age-related decline in optic atrophy 1 (OPA1), a mitochondrial protein, leading to mitochondrial dysfunction; finally, it results in muscle loss. Specifically speaking, the ablation of Opa1 results in endoplasmic reticulum stress, which signals via the FoxOs and unfolded protein response, inducing a catabolic program of systemic aging and muscle loss (Tezze et al., 2017). Interestingly, evidence shows that there is a strict connection between microbiota and mitochondrial function. There is a regulatory relationship between the gut microbiota and mitochondria; therefore, a disruption of the relationship leads to dysfunctional mitochondria. A network analysis shows that *Atopobium parvulum* was the most prominent microbe associated with mitochondrial dysfunction and the relative abundance of *A. parvulum* correlates negatively with both the level of butyrate producers and the mitochondrial protein expression. Besides, the loss of butyrate producers will cause decreased butyrate production that in turn will impair mitochondrial functions (Mottawea et al., 2016). What is more, SCFAs are the putative mediators of the effect of gut microbiome on skeletal muscle, whose main host targets are skeletal muscle mitochondria; it also suggests a close connection between microbiota and mitochondrial function (Kimura et al., 2014; Clark and Mach, 2017).

**FIGURE 3:**
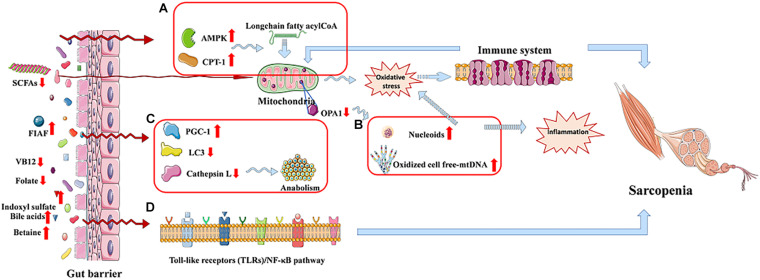
The gut–muscle axis led to sarcopenia via regulating the processes of mitochondrial dysfunction and modulation of host gene expression. **(A)** AMP-activated protein kinase (AMPK) and carnitine palmitoyl transferase-1 (CPT-1) are increased. **(B)** Mitochondrial dysfunction. **(C)** Autophagy-lysosomal pathway related proteins reduced. **(D)** Cell signaling pathway regulation process.

It is worth noting that mitochondrial dysfunction may play a vital role to link the relation between chronic inflammation and age-related sarcopenia, and the gut microbiota dysbiosis may be a key role in the gut–muscle crosstalk (Picca et al., 2018a). Nucleoids or oxidized cell free-mtDNA extruded from damaged mitochondria could trigger inflammation in sarcopenia (Figure 3B); they would activate the innate immune system as well as induce body to produce inflammatory mediators. The release of the latter would maintain a vicious circle in myocytes, leading to further mitochondrial impairment, finally resulting in sarcopenia (Picca et al., 2018b). Moreover, mitochondrial dysfunction is suggested as one of the possible mechanisms for anabolic resistance (Ni Lochlainn et al., 2018).

### Modulation of Host Gene Expression

In fact, gut microbiota is able to produce a variety of metabolites that reach the muscle to give proanabolic signals to the host, modulating gene expression of the host and enhancing antioxidant activity.

Bäckhed et al. show that the activities of AMP-activated protein kinase, which monitor cellular energy status, as well as carnitine palmitoyl transferase-1, are increased in the muscle of GF mice compared to mice with gut microbiota, promoting long-chain fatty acyl-CoA to enter into mitochondria, where they will be oxidized, which indicates an increased oxidative capacity (Figure 3A). Besides, GF mice show increased intestinal levels of fasting-induced adipocyte factor that is associated with an increased expression of proliferator-activated receptor-γ coactivator-1 in the gastrocnemius muscle. These increased activities counteract the effect of denervation and fasting on muscle atrophy (Backhed et al., 2007; Bindels and Delzenne, 2013; Picca et al., 2018a). A study shows a reduction of LC3 and cathepsin L in tibialis and gastrocnemius muscles, and they are two markers of the autophagy–lysosomal pathway, which is a major system, in skeletal muscle, of protein breakdown (Figure 3C; Mammucari et al., 2007; Bindels and Delzenne, 2013). It is reported that a healthy gut microbiota can produce a mass of complex cobalamin (or vitamin B_12_) and folate, which may prevent reduction of muscle function through improving muscle anabolism, as well as preventing both hyperhomocysteinemia-induced oxidative stress and endothelial damage (Kuo et al., 2007; Guy LeBlanc et al., 2013). Besides, indoxyl sulfate, gut microbiota-specific metabolite, can lead C2C12 myoblasts to increase the activity of the pentose phosphate pathway and show an up-regulation of glycolysis, inducing sarcopenia (Wikoff et al., 2009; Sato et al., 2016; Grosicki et al., 2018). In addition, bile acids produced by gut microbiota can be signaling molecules in regulating G-protein–coupled bile acid receptor-1 (also known as TGR5) in the muscle, promoting intracellular thyroid hormone activation, and thereby elevate energy expenditure in human skeletal muscle cells (Watanabe et al., 2006; Di Ciaula et al., 2017). Except this, bile acids also prevent muscle fat deposition by activating the nuclear farnesoid X receptor (Watanabe et al., 2006; Cipriani et al., 2010; Di Ciaula et al., 2017). Nuclear factor κB (NF-κB), the transcription factor of muscle-specific activation, causes sarcopenia, which calls toll-like receptors/NF-κB pathway (Figure 3D), and may be associated with the gut microbiota-muscle axis (Bindels and Delzenne, 2013). What is more, betaine is a microbial metabolite that activates cytosolic calcium influx, synthesis of IGF-1, and extracellular signal–regulated kinase in human osteoblast cultures, which may have effects on skeletal muscle (Villa et al., 2017; Grosicki et al., 2018). The induction of Klf15 gene expression and activation of the branched-chain amino acid pathway in GF mice may be another possible mechanism of gut–muscle axis, which is called HPA-glucocorticoid–driven atrophy of skeletal muscle mass (Lahiri et al., 2019).

### Others

Interestingly, gut microbiota could also affect muscle function via the mediation of the central nervous system (Ticinesi et al., 2017), Lahiri et al. observed reduced serum choline, which is the precursor of acetylcholine and the key neurotransmitter that signals between muscle and nerve at neuromuscular junctions (NMJs) in GF mice, which suggests that the communication between muscle and nerve cells at NMJs may be impaired under GF conditions. Besides, gut microbiota is observed to alter the expression of genes encoding Rapsyn and Lrp4, two proteins important for NMJ assembly and function expression of NMJ-associated genes in GF mouse muscle alterations (Lahiri et al., 2019). However, further experiments are needed to reach a definitive conclusion.

## Treatment

On the basis of mechanisms of gut–muscle axis, a significant amount of studies suggest that gut microbiota modulation may potentially be a future therapeutic target in age-related sarcopenia. In this review, we will summarize the possible ways to modulate gut microbiota and thereby come up with potential treatments of sarcopenia.

### Modulate Gut Microbiota Directly

#### Supplement of Probiotics and Prebiotics and Dietary Fibers

More and more animal and human studies suggest that the use of prebiotics and/or probiotics has beneficial effects on skeletal muscle. Prebiotics, fermented in the lower part of the gut and selectively stimulating the growth and/or activity(ies) of a limited number of bacteria, are non-digestible carbohydrates and therefore have healthy effects on the host (Delzenne and Cani, 2011). There are two substances, inulin and *trans*-galacto-oligosaccharides, meeting the criteria for classification as a prebiotic (Steves et al., 2016; Vandeputte et al., 2017), and more investigated prebiotics anticipated as standard in recent years (Jäger et al., 2019; Khuituan et al., 2019). Cani et al. (2009) demonstrate that the supplement of prebiotics has beneficial efficacy on skeletal muscle of mice. Besides, an increase in muscle strength and endurance can be observed after prebiotic supplementation (inulin plus fructo-oligosaccharides) in older people (Buigues et al., 2016). Probiotics are live microorganisms, such as *Lactobacillus* species and so on, which confer a health benefit on the host when administered in adequate amount (Delzenne and Cani, 2011). Sarcopenia is attenuated through oral supplementation with specific *Lactobacillus* species in a mice model of acute leukemia (Bindels et al., 2012). Besides, the muscle mass and function increased by supplementation with *Lactobacillus plantarum* (Chen et al., 2016). Treatment with probiotic formulations, containing *Faecalibacterium prausnitzii*, which is one of the main SCFA producers, is associated with reduced systemic inflammation in mice (Munukka et al., 2017). As for human studies, two probiotic trials show an increase in skeletal muscle function in elite athletes (Salarkia et al., 2013; Shing et al., 2014). Interestingly, it is suggested that supplement of prebiotic and/or probiotic elevates the abundance of butyrate producers, as well as *Bifidobacterium*, and thereby improves the muscle mass and function in older people (Vulevic et al., 2008; Eloe-Fadrosh et al., 2015). However, most of the evidence for a benefit of probiotics is in rodents. In humans, probiotic studies have only shown evidence in randomized controlled trials in young populations or very old or the severely ill (Steves et al., 2016). Moreover, dietary fibers play a key role in gut microbiome diversity and composition, and its supplementation may be a significant approach for increasing gut bacterial SCFA production (Lustgarten, 2020), which may have positive effects on skeletal muscle mass and physical function in humans (Lustgarten, 2019).

### Fecal Microbiota Transplantation

Fecal microbiota transplantation (FMT) is the infusion of a solution of donor feces to patients (van Nood et al., 2013). Many studies suggest that FMT could potentially be a method to improve skeletal muscle mass and function. Yan et al. (2016) transfer gut microbiota from obese pigs to GF mice, and the result is that the metabolic profile and fiber characteristics of the skeletal muscle are replicated in the recipients, which indicates the beneficial effect of FMT on skeletal muscle performance. Compared to supplementation with prebiotic and/or probiotic, FMT is a more radical treatment option (Steves et al., 2016).

### Diet and Nutrition

During aging, most of gut microbiota alterations due to diet, in other words, gut microbiota composition, depends heavily on diet composition (Claesson et al., 2012). It is suggested that long-term dietary habits have important effects on gut microbiome (Fielding et al., 2019); according to the report, increased levels of *Prevotella*, some *Firmicutes*, and SCFAs are significantly associated with consumption of a Mediterranean diet (De Filippis et al., 2016). In fact, except changing the composition of gut microbiota, nutrition may also influence metabolic process of it, such as modulating the host gene expression via various mediators, because most of the mediators produced by gut bacteria originate from dietary intake (Salazar et al., 2017). In this context, new nutritional therapeutic avenues have been proposed to relieve age-related sarcopenia (Siddharth et al., 2017). We will summarize the balance effects of different nutrients supplements on the gut and/or skeletal muscle health of animal models or patients in Table 1.

**TABLE 1:** The balance effects of different nutrients supplements and exercise on the gut and/or skeletal muscle health of animal models or human.

Methods	Objects	Effects	Possible mechanisms involved	References
Prebiotics supplement: *oligofructose*	Mice	Reduction of intestinal permeability and improvement of tight-junction integrity	Improved gut barrier functions through glucagon-like peptide-2-dependent mechanism (GLP-2) and reduced inflammatory tone	Cani et al., 2009
Prebiotics supplement: *a mixture of inulin plus fructo-oligosaccharides*	Older people	Improvement of muscle strength	Reduced proinflammatory cytokines in blood and inflammation	Buigues et al., 2016
Prebiotics supplement: *GOS mixture (B-GOS)*	Older people	Decrease of less beneficial bacteria and an increase of beneficial bacteria	Improved NK cell activity and phagocytosis, increased secretion of the anti-inflammatory cytokine interleukin 10 (IL-10), and decreased secretion of proinflammatory cytokines [IL-6, IL-1β, and tumor necrosis factor (TNF-α)]	Vulevic et al., 2008
Probiotics supplement: *L. reuteri100-23 and L. gasseri 311476*	Mice	Increase of tibialis muscle weight	Decreased the plasma level of inflammatory cytokines (IL-4, monocyte chemoattractant protein 1, IL-6, granulocyte colony-stimulating factor) and maintained the plasma level of the anti-inflammatory IL-10, to modulate inflammation and systemic immunity	Bindels et al., 2012
Probiotics supplement: *L. plantarum* TWK10 (LP10)	Mice	Increase of muscle mass, strength and endurance	Reduced inflammation; enhanced glucose utilization by increasing the number of gastrocnemius type I muscle fibers	Chen et al., 2016
Probiotics supplement: *Faecalibacterium prausnitzii*	Mice	Increase of muscle mass	Enhanced mitochondrial respiration; improved intestinal integrity and reduced inflammation; improved insulin sensitivity	Munukka et al., 2017
Probiotics supplement: probiotics capsule	Athletes	Increase of muscle endurance	Reduction in gastrointestinal permeability, serum LPS concentrations and inflammation	Shing et al., 2014
Probiotics supplement: *Lactobacillus rhamnosus GG ATCC 53103 (LGG)*	Older people	Modified the resident microbiota, producers of the short-chain fatty acid (SCFA) butyrate increased	Reducing intestinal permeability and inflammation by SCFA butyrate	Eloe-Fadrosh et al., 2015
Fecal microbiota transplantation (FMT)	Pig and mice	Transfer of fiber characteristics and lipid metabolic profiles of skeletal muscle from pigs to germ-free mice through gut microbiota	Specific gut microbiome could inhibit ectopic fat deposition in skeletal muscle and enhances muscle growth	Yan et al., 2016
Protein supplement	Human	Improvement of muscle mass and function	Increase of overall diversity of gut microbiota that can alter the bioavailability of amino acids, influencing muscle protein synthesis and breakdown	Bechshoft et al., 2016; Singh et al., 2017; Karlund et al., 2019
Dietary fiber supplement	Mice	Increase of muscle mass and strength	Fecal SCFAs increased, improving intestinal barrier function and reducing inflammation	Lustgarten, 2019, 2020 13,14
SCFAs (butyrate) supplement	Mice	Increase of muscle fiber cross-sectional area and prevention of intramuscular fat accumulation	Butyrate is a histone deacetylase (HDAC) inhibitor; HDACs regulate myogenesis via the transcription factor myocyte enhancer factor−2	Walsh et al., 2015
Exercise	Human	Increase of ability and capacity of skeletal muscle to synthesize proteins	Increased gut microbiota biodiversity and representation of taxa with beneficial metabolic functions, and gut microbiota could conversely have influence on muscle through modulating the inflammatory response, changing bioavailability of dietary proteins and producing substances that have beneficial and proanabolic effects in skeletal muscle cells; fecal SCFAs increased, improving intestinal barrier function and reducing inflammation	Dhillon and Hasni, 2017; Lustgarten, 2019; Ticinesi et al., 2019a

### High-Protein Diets

A study shows that protein consumption is correlated positively with gut microbiota diversity (Singh et al., 2017), and the source of protein seemed influential. In an animal study, compared to animal protein feeding, hamsters show higher microbial diversity in those fed soy protein (An et al., 2014; Butteiger et al., 2016). As is known to all, supplementation with protein can improve skeletal muscle mass and function, and it has been reported that a high-protein diet (HPD) adhering to the recommended acceptable macronutrient distribution ranges might help reduce sarcopenia (Ford et al., 2020). Moreover, studies suggest that whey protein and leucine supplementation appeared to improve muscle mass and function (Bechshoft et al., 2016; Kobayashi, 2018). However, further study is needed to prove that the increase in muscle mass and function through HPDs is involved in the modulation of gut microbiota.

### Supplement of Other Nutrients and Plant Active Substances

Recently, an animal study shows that feeding a mixture of SCFAs to GF mice was able to improve muscle strength (Lahiri et al., 2019). In addition, there is evidence showing that butyrate treatment may be a promising approach to counteract age-related sarcopenia; by the way, butyrate can be produced by *Bifidobacterium* (Walsh et al., 2015). Melatonin is proved to have the activity of effectively improving age-related skeletal muscle disorders (Stacchiotti et al., 2020). It possibly targets mitochondria through the gut–muscle axis, effectively maintaining mitochondrial function, scavenging free radicals, reducing oxidative damage, and achieving the purpose of maintaining age-related sarcopenia. Notably, plant active substances have also been confirmed to have an impact on gut and skeletal muscle health. A recent study shows that lycopene has a dose-dependent health effect on the human intestine (Wiese et al., 2019). The relative abundance of *Bifidobacterium* in the adolescent group and *Bifidobacterium longum* in the lycopene-treated middle-aged group increases and is accompanied by dose-dependent changes in blood, liver metabolism, and skeletal muscle and skin parameters. Additionally, RSV, a polyphenol found in walnuts, berries, and grapes, is capable of improving muscle performance in mature aged mice (Rodriguez-Bies et al., 2016). After 4 months of RSV treatment for middle-aged men with metabolic syndrome, it was found that the composition of the intestinal microflora is changed, accompanied by an increase in biochemical indicators of skeletal muscle turnover process (Korsholm et al., 2017). Dietary nutrients and natural plant active substances commonly have thriving biological activity and biocompatibility, while they are often required for the gut microbiome as a medium for health function. Therefore, it is of great significance to investigate their interventional and improvement effects in diseases, such as age-related sarcopenia, through the gut–muscle axis. However, further studies are needed to prove the effect of the supplement of these nutrients and plant active substances.

### Exercise

There are no pharmacologic agents for the treatment of sarcopenia, and the main treatment of sarcopenia is physical therapy for muscle strengthening and gait training (Dhillon and Hasni, 2017). Exercise training (particularly resistance training) has long been regarded as the most prospective method for improving muscle mass and strength in older people (Giallauria et al., 2016), and almost all clinical trials have proved the beneficial effects of exercise in preventing sarcopenia (Beaudart et al., 2017). Exercise has significant impact on intestinal microbiome, because some investigations show that exercise is associated with increased biodiversity, as well as representation of taxa with beneficial metabolic functions (Clarke et al., 2014; Bressa et al., 2017; Ticinesi et al., 2019a). It is suggested that there is a bidirectional relationship between skeletal muscle and the gut microbiome (Ni Lochlainn et al., 2018). Interestingly, a large number of studies suggest that there is a synergistic effect in improving muscle performance, between exercise and supplementation with protein (Bechshoft et al., 2016; Kobayashi, 2018; Ni Lochlainn et al., 2018). However, the findings of using a combined approach of exercise and dietary protein supplements were inconsistent among various populations; thus, further studies are needed (Dhillon and Hasni, 2017).

## Discussion

In conclusion, gut microbiota plays a highly important role in skeletal muscle, and chronic inflammation is the main pathogenic mechanism of sarcopenia. In fact, systemic chronic inflammation also represents the substrate of aging and a significantly important risk factor for both morbidity and mortality in older people (Picca et al., 2018a). However, inflammation-independent changes in skeletal muscle cell metabolism have also been observed, such as autophagy–lysosomal pathway, which illustrates that gut microbiota may affect the skeletal muscle mass and strength through multitude of ways. The potential therapeutic methods targeting gut microbiota are various accordingly, but not all methods work in all situations. For example, the treatment of exercise is not suitable for someone bedridden, such as paralyzed old people. The good news is that more and more studies are exploring the mechanisms of gut–muscle axis, and the new potential therapeutic approaches will be put forward accompanied by the breakthrough of the discovery of mechanism of gut–muscle axis, highlighting the multitude of ways in which gut microbiota may influence the metabolic function of skeletal muscle. Notably, nutrient supplementation is one of the effective treatment methods due to its simpleness and easy realization; it has become one of the potential and promising therapeutic methods. Nutrients are capable of changing the composition of the gut microbiome and affecting the metabolic process, because most of the mediators produced by intestinal bacteria come from dietary intake (Salazar et al., 2017). The intervention of nutrients and plant active extracts on age-related sarcopenia has been considered as a new research field.

## Author Contributions

All the authors listed have made a rational and substantial contribution to the work and approved the publication.

## Conflict of Interest

The authors declare that the research was conducted in the absence of any commercial or financial relationships that could be construed as a potential conflict of interest.
